# Weekly oral alendronate in mevalonate kinase deficiency

**DOI:** 10.1186/1750-1172-8-196

**Published:** 2013-12-20

**Authors:** Luca Cantarini, Antonio Vitale, Flora Magnotti, Orso Maria Lucherini, Francesco Caso, Bruno Frediani, Mauro Galeazzi, Donato Rigante

**Affiliations:** 1 Interdepartmental Research Center of Systemic Autoimmune and Autoinflammatory Diseases, Rheumatology Unit, Policlinico Le Scotte, University of Siena, Siena, Italy; 2 Institute of Pediatrics, Università Cattolica Sacro Cuore, Rome, Italy

**Keywords:** Mevalonate kinase deficiency, Autoinflammatory disorder, Alendronate

## Abstract

**Background:**

Mevalonate kinase deficiency (MKD) is caused by mutations in the *MVK* gene, encoding the second enzyme of mevalonate pathway, which results in subsequent shortage of downstream compounds, and starts in childhood with febrile attacks, skin, joint, and gastrointestinal symptoms, sometimes induced by vaccinations.

**Methods:**

For a history of early-onset corticosteroid-induced reduction of bone mineral density in a 14-year-old boy with MKD, who also had presented three bone fractures, we administered weekly oral alendronate, a drug widely used in the management of osteoporosis and other high bone turnover diseases, which blocks mevalonate and halts the prenylation process.

**Results:**

All of the patient’s MKD clinical and laboratory abnormalities were resolved after starting alendronate treatment.

**Conclusions:**

This observation appears enigmatic, since alendronate should reinforce the metabolic block characterizing MKD, but is crucial because of the ultimate improvement shown by this patient. The anti-inflammatory properties of bisphosphonates are a new question for debate among physicians across various specialties, and requires further biochemical and clinical investigation.

## Background

The so-called “mevalonate pathway” produces the precursors of several biomolecules, as well as crucial end-products for functions ranging from cellular signalling and membrane integrity to protein prenylation and glycosylation. Many enzymes serve as catalysts along this multistep pathway and its various branches, and several inhibitor drugs target some of these enzymes, including the group of bisphosphonates, which inhibit farnesyl diphosphate synthase, usually prescribed to prevent bone resorption [1]. Alendronate is a second-generation nitrogen-containing bisphosphonate used for the treatment of metabolic bone disorders, osteoporosis, and Paget’s disease [2,3]. A rare hereditary autoinflammatory disorder, involving the second enzyme in the mevalonate/cholesterol pathway, is mevalonate kinase deficiency (MKD), caused by mutations in the *MVK* gene encoding the enzyme mevalonate kinase [4]. This disease, characterized by recurrent episodes of fever that begin during infancy and usually improve in late adolescence or adulthood, is now considered along a spectrum ranging from a milder form, called hyper-immunoglobulinemia D (or HIDS for short), to the most severe form, called mevalonic aciduria (or MVA) [5]. HIDS was first reported when high serum IgD levels and large numbers of plasma cells with cytoplasmic IgD were discovered in the bone marrow of Dutch patients with recurrent fever episodes, while MVA was the first defect recognized in the cholesterol pathway. Recently, biochemists have obtained *in vivo* and *in vitro* models of MKD by treating mouse cells with alendronate and other aminobisphosphonates, inhibiting an enzyme located at a point further down the pathway than mevalonate kinase [6,7]. These models suggest that alendronate should *increase* the biochemical abnormality, and should thus be contraindicated in patients with MKD. But this did not seem to be the case with our young patient.

### Case report

In July 2012, a 14-year-old boy was admitted to our Unit with a long history of recurrent high fevers up to 40°C (at least twice a month), combined with abdominal pain, diarrhea, vomiting, aphthous stomatitis, erythematous pharyngitis and, sometimes, chest pain. His symptoms had begun in early infancy (at about 6 months). His first fever attacks were also characterized by a remitting pustular rash all over his body; in general, the attacks lasted around 5–6 days. Over the past 13 years, his fevers had been treated with betamethasone up to 4 mg/daily, sometimes in combination with ibuprofen (8 mg/kg/dose). At age 11, the boy had developed three bone fractures in a 3-month-period, caused by minor traumas or normal daily activities, involving the right wrist, the distal phalanx of the right thumb and the distal phalanx of the left big toe.

During our first clinical examination, the boy was alert. His temperature was normal, as was his blood pressure and general physical examination. His growth had been normal, and he was between the 25^th^ and 50^th^ percentile in height and weight for age. Evaluation of antibodies in a non-symptomatic period showed increased IgD (184 IU/ml, n.v. <100 IU/ml) and normal IgA. Laboratory investigations performed during a febrile attack showed: erythrocyte sedimentation rate (ESR): 51 mm/h (n.v. <25), C-reactive protein (CRP) 1.3 mg/dl (n.v. <0.5), serum amyloid-A (SAA) 130.8 mg/L (n.v. <10), and slight proteinuria (0.36 g/24 hours). A full diagnostic work-up, including serologic tests, complement fractions, and anti-nuclear autoantibody tests was negative. A metabolic screening during the fever attack showed increased mevalonic acid in the urine (15.6 mmol/mol creatinine, n.v. <1), while bone marrow aspiration performed during a hospital stay ruled out any blood diseases.

After providing a written consent for genetic testing, the boy was tested for mutations in the *MEFV* gene, the *TNFRSF1A* gene, and the *MVK* gene, to check for familial Mediterranean fever, tumor necrosis factor receptor-associated periodic syndrome, and MKD. A homozygous V377I *MVK* mutation was found, confirming the diagnosis of MKD; the other genetic analyses were negative.

Due to his history of bone fractures and long-term use of betamethasone administration, leading to a cumulative steroid dose of about 3.5 g administered in the past 13 years, we performed bone dual-energy X-ray absorptiometry (DEXA) to evaluate bone mineral density (BMD). The test showed severe osteoporosis at the femoral necks (Z-score −3.1) and lumbar spine (Z-score −3.1) according to World Health Organization standards. After ruling out other possible causes of secondary osteoporosis, i.e. endocrine disorders, celiac disease, inflammatory bowel diseases, and sarcoidosis, the patient was started on oral alendronate (70 mg/week) in order to improve his skeletal status and potentially avoid further bone fractures.

Surprisingly, for the first time in his life, the boy had no further febrile attacks and no inflammatory episodes over the following nine months, and needed no other treatment in that period. In addition, SAA levels, checked monthly, returned to normal. He even received a flu vaccination with no flare-up of fever.

Since the boy’s mutation is regarded as a low-penetrance one [8], his evolution towards remission of MKD was thought to be spontaneous, and by mutual agreement with his parents, alendronate therapy was withdrawn. Strikingly, four weeks later, a typical MKD fever attack occurred, and alendronate was subsequently resumed during a prolonged fever episode, leading to the resolution of fever. To date, there is no sign of disease relapse four months after having restarted weekly alendronate therapy.

## Discussion

Most of our knowledge about the mevalonate pathway is currently focused on the cholesterol synthesis branch, the target of cholesterol-lowering statin drugs; less is known about the function and regulation of the non-cholesterol-related branches. MKD, which is caused by the interruption of the mevalonate/cholesterol biosynthetic pathway, remains an obscure disease, with recurring fever attacks triggered mainly by vaccinations, infections, emotional stress, trauma and surgery, combined with varying other symptoms including abdominal signs, arthralgia, lymphadenopathy, and heterogeneous skin lesions [9,10]. A clear biological understanding of the inflammatory attacks that occur in MKD is still lacking, and macropahage activation syndrome has been reported as a potential complication of MKD febrile flare [11]. Isoprenoids including farnesyl- and geranylgeranyl-pyrophosphate play a key role in the prenylation of many proteins involved in cytoskeletal functions and vesicular trafficking [12]: indeed, a deficiency of isoprenoids may trigger interleukin-1β (IL-1β) production by peripheral blood mononuclear cells, while these same cells, drawn from MKD patients exposed to geranylgeranyl pyrophosphate, produce relatively little IL-1β [13,14].

To date, more than 170 *MVK* gene variants have been described, though only a portion of these are thought to be clearly disease-causing [15]. No definite therapy exists for MKD, and no studies have been performed to establish evidence-based treatment strategies. Many patients may benefit from on-demand administration of non-steroidal anti-inflammatory drugs and corticosteroids, especially at high doses, but these treatments bring the risk of severe side effects, including meta-steroidal osteopenia or osteoporosis after prolonged use. Anti-IL-1 or anti-tumor necrosis factor-α biological agents are an effective alternative to high doses of corticosteroids in selected patients, although their drawbacks include increased risk of secondary infections and high costs [16].

We treated our patient with alendronate, a nitrogen-containing bisphosphonate, in an attempt to counteract the glucocorticoid-related severe reduction of BMD, even though *in vitro* studies had shown that bisphosphonates could worsen fever attacks in MKD by further inhibiting a metabolic pathway that was already pathologically interrupted [17]. This treatment brought about an unexpected remission of all MKD pathological symptoms and laboratory abnormalities. We then decided to stop alendronate in order to determine whether the improvement of the MKD clinical picture was in fact spontaneous, but we observed a disease relapse after four weeks. The resumption of alendronate during a fever attack ended the attack in only 2 days, and a stable disease remission was maintained at a 4-month follow-up.

In the past, alendronate has been reported to have both pro-inflammatory and anti-inflammatory effects. Deng and colleagues found that alendronate increases IL-1β in tissues (liver, spleen, and lung), but, strangely, not in the blood [18]. Later, Shikama and colleagues explained this phenomenon when they discovered that alendronate directly stimulates macrophage cells to produce pro-IL-1β, though the release of mature IL-1β was undetectable in consequence of a poor activation of caspase-1, the IL-1 converting enzyme which yields the bioactive IL-1β [19]. Bisphosphonates have also demonstrated specific anti-inflammatory effects, inhibiting macrophage nitric oxide production in a dose-dependent manner [20]. In particular, nitrogen-containing bisphosphonates seem to induce a significant reduction of synovial lining macrophage cells, resulting in a localized decreased expression of adhesion molecules in the synovial space of patients with rheumatoid arthritis [21]. The induction of apoptosis by different bisphosphonates may have a central role in explaining their anti-inflammatory effects – in fact, MKD patients’ lymphocytes have shown a defective apoptosis during fever remission, explaining the risk of overblown inflammation after minor triggers [22]. At last, alendronate has also exhibited a dose-dependent inhibition of the production of IL-1β by activated monocytes [23], although further studies are required to confirm the inhibitory effect of this agent on cytokine production and its consequences on bone resorption.

In conclusion, as bisphosphonates inhibit a metabolic pathway already pathologically interrupted in MKD patients, theoretically they should be contraindicated in these subjects. Nevertheless, alendronate anti-inflammatory effects should have prevailed in our patient giving rise to the resolution of his periodic febrile attacks. We honestly cannot explain this result at a pathogenetic level, and further *in vivo* and *in vitro* studies should be performed to clarify the exact role of bisphosphonates on MKD. Functional studies are ongoing in our laboratories. The debate is open about the potential use of a safe, inexpensive, and easily administered treatment that should control the exasperating recurrent inflammatory attacks of MKD patients.

## Competing interests

The authors declare that they have no competing interests.

## Authors’ contributions

LC, AV and DR wrote the manuscript. FM, OML, FC, BF and MG were also involved in the management of this patient: they revised and approved the final version of the manuscript.
